# Banana Cultivar Field Screening for Resistance to *Fusarium oxysporum* f.sp. *cubense* Tropical Race 4 in the Northern Territory

**DOI:** 10.3390/jof7080627

**Published:** 2021-08-01

**Authors:** Sharl J. L. Mintoff, Tuan V. Nguyen, Chris Kelly, Samantha Cullen, Mark Hearnden, Robert Williams, Jeffrey W. Daniells, Lucy T. T. Tran-Nguyen

**Affiliations:** 1 Department of Industry, Trade and Tourism, Northern Territory Government, Darwin, NT 0801, Australia; Chris.Kelly@nt.gov.au (C.K.); Samantha.Cullen@nt.gov.au (S.C.); Mark.Hearnden@nt.gov.au (M.H.); bob.williams953@bigpond.com (R.W.); Lucy.Tran-Nguyen@nt.gov.au (L.T.T.T.-N.); 2 Department of Agriculture and Fisheries, Queensland Government, Brisbane, QLD 4102, Australia; Tuan.Nguyen@daf.qld.gov.au; 3 Department of Agriculture and Fisheries, Queensland Government, South Johnstone, QLD 4859, Australia; Jeff.Daniells@daf.qld.gov.au

**Keywords:** panama disease, tropical race 4, TR4, banana resistance, banana screening

## Abstract

*Fusarium oxysporum* f.sp. *cubense*, causal agent of Panama disease, is one of the biggest threats to global banana production, particularly the Cavendish competent tropical race 4 (*Foc* TR4). It continues to spread globally with detections occurring in regions of the Middle East and new continents such as Africa and South America in the last decade. As the search was on for new management strategies and resistant cultivars to combat the disease, a banana cultivar-screening trial took place in the Northern Territory of Australia, which examined the responses of 24 banana cultivars to the soil borne fungus. These cultivars included material from TBRI, FHIA and selections from Thailand, Indonesia and Australia and evaluated for their resistance to tropical race 4 for two cropping cycles. Several cultivars displayed considerable resistance to *Foc* TR4, including several FHIA parental lines and hybrids, the Cavendish (AAA) selections GCTCV 215 and GCTCV 247 from TBRI and an Indonesian selection CJ19 showed either very little to no plant death due to the disease.

## 1. Introduction

Fusarium wilt of banana (FWB), also known as Panama disease, caused by the soil borne vascular wilt pathogen *Fusarium oxysporum* f.sp. *cubense* (*Foc*), poses a significant economic threat to banana production worldwide with its continued spread into banana growing regions. This pathogen is not new to global banana production, as symptomology of *Foc* was first described in 1876 [1]. In the presence of a susceptible host plant, pathogen infection occurs through the lateral roots, from there the pathogen moves into the rhizome via the vascular tissue where the characteristic brown-maroon vascular discoloration can be observed as the infection progresses [2,3]. As the infection spreads into the outer leaf sheaths of the pseudostem, wilting and yellowing of the oldest leaves occurs, which will eventually progress to the younger leaves leading to the death of the plant [4,5]. The genetic diversity of *Foc* enabled this pathogen to infect a diverse range of bananas, and has been broadly categorised into four races based on their pathogenicity on particular banana cultivars [6,7].

In the first half of the 20th century, *Foc* Race 1 ravaged production of Gros Michel, the standard export cultivar, which is highly susceptible. Other cultivars susceptible to *Foc* Race 1 include Lady Finger (AAB, Pome), Ducasse (ABB, Pisang Awak) and Sugar (AAB, Silk) [8,9]. In the mid-20th century, the global export trade transitioned to cultivars of the *Foc* Race 1 resistant Cavendish subgroup, and the threat of FWB virtually disappeared. *Foc* Race 2 has a banana cultivar host range that overlaps with Race 1, and is noted for its ability to infect the cultivars of the Bluggoe subgroup (ABB) and closely related cooking bananas [8,9,10]. *Foc* Race 3, is no longer considered a banana pathogen as it only appears to infect *Heliconia* sp. [6,9,10].

*Foc* Race 4 can infect cultivars in the Cavendish subgroup as well as an overlapping range of other bananas affected by Race 1 and 2. Initially *Foc* Race 4 was found affecting Cavendish plants in cooler subtropical climates or in plants suffering from some form of stress. This particular race is now referred to as *Foc* subtropical race 4 (SR4) [9,10]. However, in the 1990s, reports began to emerge of Panama disease FWB in Cavendish plantations in Southeast Asia [11,12]. The significance of these outbreaks was due to the rise of a more aggressive *Foc* race strain that could cause severe disease in Cavendish plants even under optimal growing conditions in the tropics. This more aggressive race strain was able to infect a wider range of banana cultivars, including those affected by Race 1 and 2, without a predisposing biotic or abiotic stress and was identified as belonging to Vegetative Compatibility Group (VCG) 01213/16, later known as tropical race 4 (TR4) [12,13].

Globally the pathogen has spread throughout much of Southeast Asia, as well as China, India and Pakistan, Australia, Jordan, Israel and Lebanon and a detection in Mozambique [5,12,14,15,16,17]). Recent reports indicate the presence of *Foc* TR4 in Colombia, Turkey and the island of Mayotte [18,19,20]. In Australia, *Foc* TR4 was first detected in the Northern Territory (NT) in 1997 [14]. Despite the implementation of quarantine measures in order to stop or slow the spread of the pathogen, containment of the pathogen failed and was declared endemic to the NT in 2012 [21]. In 2015, *Foc* TR4 was detected in north Queensland, a different region of Australia. This new detection was concerning since over 90% of Australia’s banana production area is located in this relatively small geographical area [22,23].

The escalating international spread of *Foc* TR4 and the significant threat it poses to global banana production and trade have focused research efforts to identify and develop *Foc* TR4 resistant cultivars. Despite the increased host range of banana cultivars affected by *Foc* TR4, there is evidence that products of some banana improvement programs have partial to full resistance and that good sources of resistance also exist amongst banana wild relatives [24,25]. The Taiwan Banana Research Institute (TBRI), for example, screens large numbers of tissue cultured Cavendish plantlets, in an attempt to identify resistant cultivars developed through somaclonal variation [26]. The names of many lines identified showing promising resistance are prefixed GCTCV (Giant Cavendish Tissue Culture Variant). Conventional breeding programs also exist such as the Fundación Hondureña de Investigación Agrícola (FHIA) where certain selected hybrids are displaying resistance to another devastating banana disease Black Sigatoka (*Pseudocercospora fijiensis*) and various races of *Foc* [27,28]. Recently, *Foc* TR4 resistant genetically modified (GM) Cavendish have been developed which show significant levels of TR4 resistance compared to non GM Grande Naine and Williams cultivars [29].

Screening banana cultivars can be achieved through large scale field trials or small scale greenhouse studies; there are advantages and disadvantages to both methods. Greenhouse studies can be used to evaluate large numbers of different banana lines in a controlled environment that is confined to a small space with the screening occurring over a short period. In some cases, pot trials can have some bearing on the disease resistance seen in the field [24,25]. However, some issues may arise through higher inoculum pressures within pots, use of younger plants; lack of a representative soil microbiome and growing conditions not representative of field conditions [30].

Field-screening trials provide the ability to screen plants in areas affected by *Foc* TR4 where resistant cultivars could be deployed. Additionally, disease assessments can be conducted over multiple crop cycles and agronomic data collected that can provide useful information on performance and potential market acceptability [30]. However, field trials can be labour intensive, take place over multiple years, be exposed to other pathogens and severe weather events [30].

We chose the field screening method in this study and not the greenhouse method due to several factors. Firstly, the ability to study new banana cultivars in an area where *Foc* TR4 is endemic. Secondly, a dedicated facility to conduct *Foc* TR4 field trials was available and allowed for accurate and replicated trials for disease resistance assessments over a long period. Thirdly, these conditions are a better reflection and relatable to what banana growers would expect to occur under field conditions over multiple cropping cycles.

In this study, we report the findings of a *Foc* TR4 resistance screening trial conducted in the Northern Territory of Australia, which included 24 banana cultivars and breeding lines assessed for their resistance to *Foc* TR4 and agronomic data collected across two cropping cycles.

## 2. Methods

### 2.1. Banana Germplasm and Growth

The tissue culture germplasm of 24 banana cultivars and breeding lines (Table 1) were provided by a Quality Banana Approved Nursery (QBAN) scheme accredited tissue culture laboratory located at the Maroochy Research Facility of the Queensland Department of Agriculture and Fisheries. The germplasm selected in this trial included four reference control cultivars of known resistance or susceptibility to *Foc* TR4 based on their performance in previous screening trials [31], FHIA-25 (Highly Resistant), FHIA-01 (Resistant), GCTCV 218 (Intermediate) and Williams (Very Susceptible). Once received the germplasm was de-flasked, potted into 100 mL pots with steam sterilised potting media, and placed in a mist house for three weeks. Plants were moved into shade tunnels to harden off nine weeks before field planting.

### 2.2. Foc TR4 Field Inoculum Preparation

The field inoculum was prepared by soaking dry Japanese millet grain (Australian Premier Seeds, Wacol, Queensland, 4076) overnight in reverse osmosis (RO) water. The millet was drained, rinsed and placed into autoclave bags at a rate of 1.5 kg soaked millet and 500 mL RO water and autoclaved twice. Once sterilised, each bag was inoculated with cultures of the *Foc* TR4 NTP-Dc 35673 (Darwin isolate identified as VCG 01213/16), that was grown until the mycelium completely covered the culture medium. Once fully grown the culture plate was cut into 1 × 1 cm squares and approximately half of the culture plate was added to the sterilised millet bags. The bag was sealed and left at room temperature (22–24 °C) for a period of 21 days and shaken every second day then stored at 4 °C until the day of planting and inoculation.

### 2.3. Trial Site Planting and Foc TR4 Inoculations

The Darwin region of the NT has a tropical savannah climate, with a distinct dry season (May–October) and wet season (November–April) [34,35]. The dry season receives little to no rain, with temperatures during the trial period ranging between 17–35 °C and an average relative humidity ranging between 35–55%. The wet season is influenced by the summer monsoons and is characterised by storm activity, rain and temperatures ranging between 23–34 °C with the relative humidity between 70–80% [34,36].

The field trial followed a randomised complete block design based on the INIBAP Technical guidelines 7 [37] and conducted at the NT Government’s Coastal Plains Research Farm (12° S, 131° E) from June 2016 to March 2018. Each cultivar contained 24 plants across four independent replicate plots (with the exception of SH-3142, due to the limited number of available plants it contained only three replicate plots). Each replicate plot comprised of six plants per cultivar. The outer plants (1 and 6) acted as borders plants whilst the internal plants (2–5) were sample plants from which data was collected. Plants were established in a single row arrangement on a raised bed, with a spacing of 1.8 m between plants in the row and an inter-row spacing of 4 m and grown using standard commercial practices for two crop cycles. All plants were inoculated with 200 mL of *Foc* TR4 colonised millet placed in each planting hole before planting as per Smith et al. 2018 [38].

### 2.4. Assessment Criteria

Disease assessments commenced fortnightly once the presence of external symptoms was observed on the very susceptible Williams cultivar. Disease criteria were based on that of Carlier et al. 2003 [37] with modifications from Walduck and Daly [39]. External symptoms were noted as leaf yellowing on the oldest leaves that was occasionally accompanied by pseudostem splitting. At plant death or harvest, the lower portion of the pseudostem was cut and examined for internal symptoms, seen as vascular browning, if no symptoms were noted, additional cuts were made at lower intervals to verify the presence or absence of *Foc* TR4 infection. The presence or absence of internal symptoms was used to assess the frequency of infection within a particular cultivar. Disease assessment data is presented as incidence, which is a proportion of the number of diseased plants within a population [40]. Agronomic measurements at different plant growth stages were used to assess and compare the performances of the selected cultivars in the presence of *Foc* TR4. The agronomic measurements collected included; date of bunch emergence, pseudostem height at bunch emergence, date of harvest and bunch weights which was based on the methodology of Carlier et al. 2003 [37]. These agronomic data was only collected for the plant crop as severe storm damage in December 2017 destroyed the trial plants three months prior to the end of the trial, preventing collection of a full first ratoon agronomic data set. The final disease assessments of these plants was conducted the day after the storm, noting the presence or absence of internal symptoms.

### 2.5. Disease Severity Ratings

In order to rate the disease severity of each cultivar, a disease scoring system was developed to differentiate the levels of disease severity observed within the field trial. The disease severity score was calculated for each cultivar in each crop cycle. This included using the number of plants containing internal disease symptoms within the pseudostem, the number of plants killed due to *Foc* TR4 infection and the total number of plants assessed for that cultivar in a particular crop cycle. The disease score was determined with the following the formula:

$$\frac{\left(\#\;plants\;with\;internal\;symptom+\#\;plants\;killed\;by\;TR4\right)\;}{\#\;assessed\;plants\;of\;cultivar\;}=Disease\;score\;\left(0-2\right)$$

The resulting scores were grouped into a resistance ratings based on their overall performance, these resistance ratings were adapted from Daniells et al. 2017 [41] (Table 2).

### 2.6. Isolation and Molecular Confirmation of Foc TR4

To confirm the presence of *Foc* TR4, at least one representative plant per replicate was sampled for each cultivar to confirm the presence of the fungus where possible. *Foc* TR4 was isolated from infected xylem tissue that was surface sterilised in 70% ethanol for one minute, rinsed in sterile distilled water and dried on dry sterile filter paper. The surface sterilised plant material was cut into smaller segments and placed onto Potato Dextrose Agar (PDA) media, with each culture plate amended with 2 drops of 25% lactic acid. The culture plates were sealed with parafilm, incubated at 25 °C and inspected 72 h later for fungal growth. The resulting *Fusarium* sp. colonies were sub-cultured and pure monoconidial culture were grown and tested using a *Foc* TR4 specific PCR assay FocTR4-F2/R1 primers (F primer 5′ CGCCAGGACTGCCTCGTGA 3′ and R primer 5′ CAGGCCAGAGTGAAGGGGAAT 3′) [42] and SIX8 (F primer 5′ TCGCCTGCATAACAGGTGCCG 3′ and R primer 5′ TTGTGTAGAAACTGGACAGTCGATGC 3′) [43].

### 2.7. Statistical Analysis

All data was analysed with R (R Core Team 2020) using the *lme4* package [44]. Data with a binomial response (internal symptoms, death attributable to *Foc* TR4) were analysed with a generalised linear mixed effects models for fixed (cultivar) and random factors (replicate blocks). Data for bunch weight, pseudostem height at bunch emergence, and data with time responses (time to bunch emergence, time to complete crop cycle and time to first appearance of external *Foc* TR4 symptoms) were analysed with linear mixed effects model for fixed (cultivar) and random factors (replicate blocks). The *emmeans* [45] and *multcomp* packages [46] were used to calculate estimated marginal means for the cultivars in each model and homogenous groups from all pairwise comparisons. All confidence levels were set at 0.95 and for the multiple comparisons, the Tukey method was used for the appropriate family of estimates keeping the Type I experiment-wise error rate at 0.05.

## 3. Results

### 3.1. Development of Disease Symptoms

The appearance of external disease symptoms was first noted in the Williams (VS) control and DPM25 approximately six months after field planting (Figure S1a). The external disease symptoms were observed as chlorosis of the leaves with eventual development of necrosis and death of the oldest leaves (Figure S2a). Confirmation of *Foc* TR4 infection was made at the plant’s death or harvest by the presence of internal symptoms observed as vascular discolouration within the pseudostem (Figure S2b).

**Figure 1:**
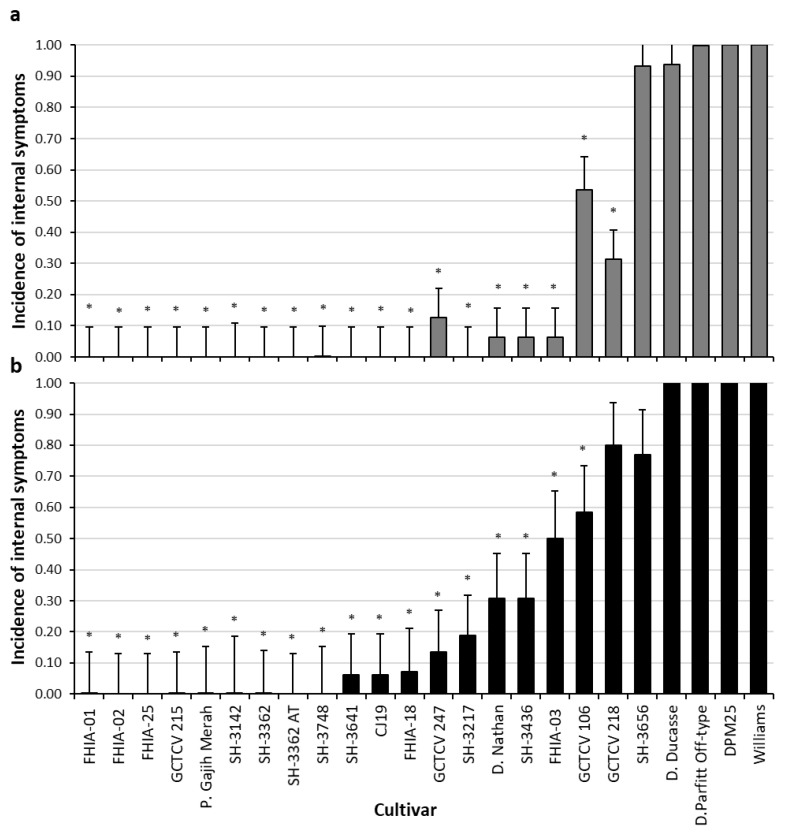
(**a**) Incidence of plants containing internal *Foc* TR4 symptoms in the plant crop cycle. (**b**) Incidence of plants containing internal *Foc* TR4 symptoms within the first ratoon crop cycle. Cultivars marked with an asterisk indicate the mean is significantly different to Williams control (*p* < 0.05). Error bars for each cultivar represent the 95% confidence interval for the mean.

The incidence of plants in the plant crop that contained internal symptoms was highest in the cultivars SH-3656, Dwarf Ducasse, Dwarf Parfitt Off-type and DPM25, and no significant difference was noted compared to the Williams (VS) control. A significantly lower incidence of plants with internal symptoms were found in the cultivars GCTCV 247, Dwarf Nathan, SH-3436, FHIA-03, GCTCV 106 and GCTCV 218 when compared to the Williams (VS) control (*p* < 0.05). No internal symptoms were noted in the cultivars: FHIA-01, FHIA-02, FHIA-25, GCTCV 215, Pisang Gajih Merah, SH-3142, SH-3362, SH-3362 (AT), SH-3748, SH-3641, CJ19 and SH-3217 (Figure 1a).

In the first ratoon crop cycle, cultivars SH-3656, Dwarf Ducasse, Dwarf Parfitt Off-type, DPM25 and GCTCV 218 all contained the highest incidence of plants with internal symptoms with no significant difference found when compared to the Williams (VS) control. A significantly lower incidence of plants possessing internal symptoms were noted in cultivars SH-3641, CJ19, FHIA-18, GCTCV 247, SH-3217, Dwarf Nathan, SH-3436, FHIA-03 and GCTCV 106 when compared to the Williams (VS) control (*p* < 0.05). No internal symptoms were noted in the cultivars: FHIA-01, FHIA-02, FHIA-25, GCTCV 215, Pisang Gajih Merah, SH-3142, SH-3362, SH-3362 (AT) and SH-3748 (Figure 1b). Isolation of the pathogen occurred from representative samples and confirmed via PCR (Figure S3).

### 3.2. Plant Death

**Figure 2:**
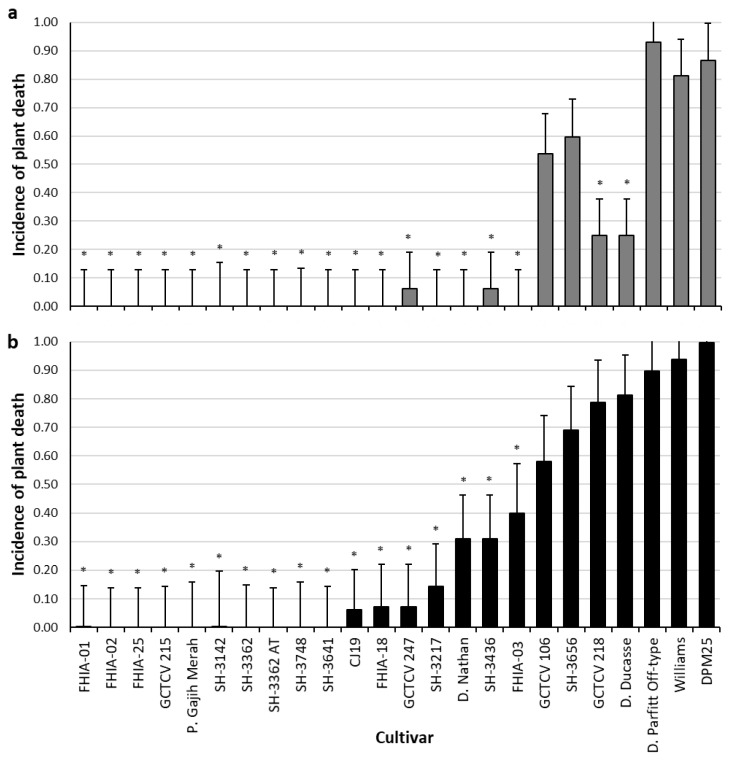
Incidence of plant death attributed to *Foc* TR4 in field trial plants within the (**a**) plant crop and (**b**) first ratoon crop. Cultivar means marked with an asterisk indicate the mean is significantly different to Williams control (*p* < 0.05). Error bars for each cultivar represent the 95% confidence interval for the mean.

Plant death attributed to *Foc* TR4 within the plant crop, were the highest in the cultivars GCTCV 106, SH-3656, Dwarf Parfitt Off-type and DPM25 with no significant difference found when compared to the Williams (VS) control. Significantly lower incidence of plant death was noted in GCTCV 247, SH-3436, GCTCV 218 and Dwarf Ducasse when compared to the Williams (VS) control (*p* < 0.05). No death caused by the pathogen was noted in the remaining cultivars FHIA-01, FHIA-02, FHIA-25, GCTCV 215, Pisang Gajih Merah, SH-3142, SH-3362, SH-3362 (AT), SH-3748, SH-3641, CJ19, FHIA-18, SH-3217, Dwarf Nathan and FHIA-03 (Figure 2a).

In the subsequent ratoon crop cycle, no significant difference in mortality was noted within the GCTCV 106, SH-3656, GCTCV 218, Dwarf Ducasse, Dwarf Parfitt Off-type and DPM25 and when compared to the Williams (VS) control. Plant death in the CJ19, FHIA-18, GCTCV 247, SH-3217, Dwarf Nathan, SH-3436 and FHIA-03 were significantly lower compared to the Williams (VS) control (*p* < 0.05). No plant death attributed to *Foc* TR4 were noted in the cultivars FHIA-01, FHIA-02, FHIA-25, GCTCV 215, Pisang Gajih Merah, SH-3142, SH-3362, SH-3362 (AT), SH-3748 and SH-3641 (Figure 2b).

### 3.3. Disease Severity

FHIA-25 (HR) and FHIA-01 (R) controls both received a score of zero, which gave them both a highly resistant rating. The GCTCV 218 (I) control was rated as intermediate within the plant crop cycle, and very susceptible in the first ratoon crop cycle as the infection rates increased. The Williams (VS) control received a rating of very susceptible in both crop cycles. Most cultivars retained their resistance rating from the plant crop in the first ratoon. The other cultivars showed an increase in susceptibility as disease symptoms or mortality became apparent in the first ratoon crop cycle (Table 3).

### 3.4. Cultivar Bunch Emergence and Plant Height

The average bunch emergence time for Williams occurred at 30.9 weeks post planting. Significantly different times of bunch emergence were found for the cultivars: FHIA-03, SH-3142, SH-3436, GCTCV 218, GCTCV 215, Dwarf Ducasse, SH-3217, which ranged from 35.5 to 40.6 weeks (*p* < 0.05). The cultivars, FHIA-25, GCTCV 106 and SH-3362 recorded the longest time to bunch emergence with average times greater than 45 weeks (45, 46.2 and 48.9 weeks, respectively) which was significantly longer than the Williams control (Table 4). The SH-3362 Auto-tetraploid did not display any signs of flowering or disease symptoms at 12 months after planting.

The cultivars Dwarf Nathan and CJ19 were the shortest cultivars measured (111 and 207 cm, respectively) and significantly shorter than the Williams control (255 cm) (*p* < 0.05). While the cultivars: SH-3641, FHIA-03, FHIA-18, FHIA-01, SH-3436, SH-3142, FHIA-25, SH-3748, SH-3362, SH-3217 and Pisang Gajih Merah (*p* < 0.05) were significantly taller compared to Williams, with the tallest plants SH-3217 and Pisang Gajih Merah displaying average heights of 356 and 408 cm, respectively (Table 4).

### 3.5. Plant Crop Cycle Times and Bunch Weights

Plant crop cycle times ranged from 39.5 to 61.2 weeks post planting across the banana cultivars tested (Table 5). Dwarf Nathan had the shortest crop cycle with an average of 39.5 weeks post planting which was significantly quicker (*p* < 0.05) compared to the Williams control (45 weeks). Significantly longer crop cycles times were noted in cultivars SH-3436, FHIA-03, FHIA-18, Pisang Gajih Merah, SH-3142 (ranging from 47.1–49.7 weeks post planting) and in GCTCV 215, Dwarf Ducasse, SH-3217, FHIA-25 and SH-3362 (ranging from 50–62 weeks post planting) (*p* < 0.05). Bunch weights varied between different cultivars and ranged from 15.9 to 35.4 kg. Dwarf Nathan had the lowest average bunch weight (15.9 kg), significantly less than Williams (*p* < 0.05). The cultivars FHIA-03, FHIA-25, SH-3436, SH-3748, Pisang Gajih Merah and FHIA-01 all possessed significantly larger bunch weights (>30 kg) compared to the Williams control (*p* < 0.05) (Table 5).

## 4. Discussion

Of the 24 cultivars tested in this trial, nine cultivars rated as highly resistant and did not succumb to *Foc* TR4 infection, with an additional four cultivars rated as resistant with minimal effects of the disease noted, which included Cavendish lines, FHIA lines and Pisang Gajih Merah. The inclusion of four reference control cultivars of known disease resistance to *Foc* TR4 provided reference points for potential comparisons to other trials. Their importance is highlighted as disease severity can vary from year to year with environmental conditions and the level of disease inoculum pressure present. The intermediate control, GCTCV 218, was chosen in particular to also act as a guide for the level of disease resistance to which a cultivar must at least attain to be considered for commercial production in TR4 infested locations.

The most susceptible cultivars were Williams (VS) control, DPM25, Dwarf Parfitt Off-type, SH-3656 and Dwarf Ducasse, which consistently possessed the highest disease scores in their plant or ratoon crop cycles. The disease severity noted in the Williams and DPM25 was consistent with previous inoculated field trials that included these particular cultivars [29,31,39]. DPM25 is a gamma irradiated mutant of the extra dwarf Cavendish cultivar ‘Dwarf Parfitt’ and was selected for its superior agronomic qualities comparable to Williams and its increased resistance to *Foc* SR4 [32]. Additionally, SH-3656 was previously screened for its resistance to *Foc* Race 1 and SR4 in the Australian subtropics where it was reported to be susceptible to *Foc* R1 and resistant to *Foc* SR4 [47]. Although DPM25 and SH-3656 had previously shown promising resistance to *Foc* SR4 in the Australian subtropics its resistance to *Foc* SR4 did not translate to *Foc* TR4 resistance.

Among the Cavendish cultivars that showed some level of resistance greater than very susceptible, the Taiwanese somaclones GCTCV 106 and GCTCV 218 (commonly known as Formosana) did not succumb so quickly. The GCTCV 106 held in the Australian germplasm collection demonstrated generally poor growth performance, vigour and pale green leaves, suggesting that may not be true to type. This is supported by indications from the Philippines that GCTCV 106 in their *Foc* TR4 field screening trials demonstrated much better agronomic performances than in this trial (A. Molina, *pers. comm*., 2021). The performance of GCTCV 218 is consistent with previous field trials conducted in the NT, where high disease incidence due to *Foc* TR4 was observed [29,31,39]. GCTCV 218 has shown some resistance to *Foc* TR4 in naturally infested field conditions, with its deployment in the Philippines, Taiwan and Mozambique as a means to minimise losses due to the wilt disease while still producing good quality bunches [5,26,48,49]. The contrasting results of GCTCV 218 between the Australian trials to those in the Asia and Africa is likely due to the higher inoculum pressure and its uniform distribution with all plants inoculated at planting in the Australian trials, leading to increased mortality and disease severity as a result.

The cultivars Dwarf Nathan, SH-3436 and FHIA-03 all shared similar levels of plant death due to *Foc* TR4 infection with a subsequent rise in plants affected by the disease in the ratoon crop cycle. While FHIA lines SH-3641 and SH-3217 and the Cavendish GCTCV 247 and CJ19 possessed a relatively low incidence of disease symptoms and death over two crop cycles. Even though these particular lines suffered some plant death in the ratoon crop, we expect that those losses would be much less in naturally infested plantations which have been appropriately managed to reduce disease inoculum pressure compared to our trial inoculations which provide uniformly high disease levels throughout.

The Cavendish somaclones, GCTCV 247, CJ19 and GCTCV 215, demonstrated little to no disease incidence during the trial period and possessed a higher level of resistance compared to other Cavendish cultivars such as GCTCV 218 and Williams. The resistance seen in the GCTCV 215 and GCTCV 247 is encouraging, as these lines have shown similar resistance in previous field and pot trials when inoculated with *Foc* TR4 [25,26]. Although the GCTCV 215 displayed significant resistance to *Foc* TR4 it also possessed a longer crop cycle time, a characteristic noted in previous research, making it the longest cycling Cavendish cultivar in this trial [26]. CJ19 (ex. Indonesia) demonstrated robust resistance to *Foc* TR4 and in the NT and did not develop severe leaf twisting noted in plantings in Far North Queensland. In a previous *Foc* TR4 field screening trial CJ19 was deemed susceptible [31]. The greater susceptibility in earlier work is likely due to exposure to a much higher inoculum level as in that same work FHIA-01 displayed disease symptoms during their trial.

The resistant and highly resistant reference control cultivars, FHIA-01 and FHIA-25, respectively, were chosen based on the earlier work of Walduck and Daly [39]. In this trial, no disease symptoms were observed for either in plant or first ratoon. In comparison to the trial of Walduck and Daly [39], FHIA-01 displayed some disease symptoms in the plant crop yet recovered in the subsequent crop cycle, which indicates the inoculum pressure was considerably higher. The resistance of the three FHIA cultivars FHIA-01, FHIA-18 and FHIA-25, may stem from their shared male parent SH-3142 [28]. Additionally, previous field screening of FHIA-01 and FHIA-18 have shown them to be resistant to *Foc* Race 1 and SR4 when tested in the Australian subtropics [50]. FHIA-02 possesses resistance to Black Sigatoka [51,52], and also showed a high level of resistance to *Foc* TR4 with no symptoms or plant death attributed to the pathogen in either crop cycle. FHIA-02 also appears to be resistant to Race 1 (VCG 0124/5) in the tropics [41]. However, field trials in the Australian subtropics have shown it can be susceptible to *Foc* Race 1 and SR4 [47], but its susceptibility to TR4 in the subtropics is not yet known. The parent line SH-3142 did not display any signs of disease in either crop cycle, and its performance is consistent with the results of previous pot trials where it did not show signs of rhizome discolouration or disease symptoms when inoculated with *Foc* TR4 [24]. Pisang Gajih Merah, a Saba type cooking banana from Indonesia, also displayed strong resistance to *Foc* TR4 with no disease symptoms noted throughout the trial. This cultivar is an extremely vigorous plant with good resistance to leaf diseases such as Black Sigatoka and its resistance to *Foc* TR4 may be beneficial in areas relying on cooking bananas impacted by TR4 [53].

This study demonstrated several cultivars possessed high levels of resistance to *Foc* TR4, with some of these representing potential parental lines for future conventional breeding endeavours. Additionally, some of the resistant cultivars could be potential candidates for future improvement via mutagenesis or genetic modification. Further studies are needed to determine the effects that different environments may have on these resistant cultivars and should include trials on commercial farms to gain grower insights on their performance over a longer period, which would be invaluable.

## Figures and Tables

**Table 1:** Banana cultivars assessed for resistance to *Foc* TR4 in this study and origin of banana germplasm.

Cultivar	Genotype	Description	Origin/Breeding Program
Williams (VS reference)	AAA	Cavendish	Australian Industry standard
GCTCV 106 ^1^	AAA	Cavendish	TBRI
GCTCV 215	AAA	Cavendish	TBRI
GCTCV 218 (I reference)	AAA	Cavendish	TBRI
GCTCV 247	AAA	Cavendish	TBRI
Dwarf Nathan	AAA	Cavendish	Israel
CJ19	AAA	Cavendish	Ex. Indonesia
Dwarf Parfitt Off-type	AAA	Cavendish	Tissue culture variant
DPM25 ^2^	AAA	Cavendish	Dwarf Parfitt Mutant
Dwarf Ducasse	ABB	Pisang Awak	Thailand
Pisang Gajih Merah	ABB	Saba	Indonesia
SH-3142	AA	Elite parent	FHIA
SH-3748	AAB	‘Cooking hybrid’	FHIA
SH-3362	AA	Elite parent	FHIA
SH-3362 Auto-tetraploid (AT)	AAAA	Ploidy modified SH-3362	Queensland DAF ^3^
SH-3641	AAAB	Pome hybrid	FHIA
SH-3217	AA	Elite parent	FHIA
SH-3436	AAAA	Highgate hybrid	FHIA
SH-3656	AAAB	Pome hybrid	FHIA
FHIA-01 (R reference)	AAAB	Pome hybrid	FHIA
FHIA-02	AAAA/AAAB ^4^	‘Dessert hybrid’	FHIA
FHIA-03	AABB	‘Cooking hybrid’	FHIA
FHIA-18	AAAB	Pome hybrid	FHIA
FHIA-25 (HR reference)	AAB,	‘Cooking hybrid’	FHIA

VS = Very Susceptible, I = Intermediate, R = Resistant and HR = Highly Resistant. ^1^ There is some uncertainty that this is the original selection made by TBRI as its TR4 disease reaction and its general overall poor vigour/pale green leaves in this trial does not match that described in Taiwan. ^2^ DPM25–a mutant originally generated as per Smith et al. 2006 [32]. ^3^ Developed as per Hamill et al. 1992 [33]. ^4^ There is some uncertainty regarding parentage. Seed were originally obtained from the fruit of a Williams Cavendish pollinated bunch, but SSR marker studies by the Musa Genotyping Centre (not presented) suggest likely AAAB genome.

**Table 2:** Disease severity scores and related resistance rating.

Disease Score	Resistance Rating	Definition
0	Highly Resistant (HR)	Plants do not show disease symptoms under high inoculum pressure.
0.01–0.3	Resistant (R)	Plants normally show no symptoms, yet can show low incidence of disease under high inoculum pressure.
>0.3–1	Intermediate (I)	Plants that can continue to grow and develop whilst showing less severe symptoms than more susceptible cultivars under natural infection conditions. The level of inoculum pressure may influence susceptibility or resistance [5].
>1–1.4	Susceptible (S)	More than 50% of plants affected. Determined by the presence of symptoms or disease mortality.
>1.4–2	Very Susceptible (VS)	Severe disease symptoms and high mortality rates due to infection, more than 70% of plants affected.

**Table 3:** Disease severity score and resistance ratings for all assessed cultivars in both the plant and ratoon crop cycles. Ratings in bold indicate a change in rating compared to the previous crop cycle.

	Plant Crop		Ratoon Crop	
Cultivar	Score	Rating	Score	Rating
FHIA-25	0.00	HR	0.00	HR
FHIA-01	0.00	HR	0.00	HR
GCTCV 215	0.00	HR	0.00	HR
FHIA-02	0.00	HR	0.00	HR
SH-3362 (AT)	0.00	HR	0.00	HR
SH-3362	0.00	HR	0.00	HR
Pisang Gajih Merah	0.00	HR	0.00	HR
SH-3142	0.00	HR	0.00	HR
SH-3748	0.00	HR	0.00	HR
SH-3641	0.00	HR	0.07	**R**
CJ19	0.00	HR	0.13	**R**
FHIA-18	0.00	HR	0.14	**R**
SH-3217	0.00	HR	0.36	**I**
GCTCV 247	0.19	R	0.21	R
FHIA-03	0.06	R	1.00	**I**
Dwarf Nathan	0.06	R	0.62	**I**
SH-3436	0.13	R	0.62	**I**
GCTCV 218	0.56	I	1.64	**VS**
GCTCV 106	1.08	S	1.17	S
Dwarf Ducasse	1.19	S	1.81	**VS**
SH-3656	1.53	VS	1.46	VS
Williams	1.81	VS	1.94	VS
Dwarf Parfitt Off-type	1.93	VS	2.00	VS
DPM25	1.93	VS	2.00	VS

**Table 4:** Bunch emergence and plant height of surviving plants with the plant crop. Error value for each cultivar is represented by the confidence interval set at 95% (CL 95%) for the mean, an asterisk indicates the mean is significantly different to Williams control (*p* < 0.05).

	Bunch Emergence				Plant Height (cm)			
Cultivar	Weeks	CL 95%			cm	CL 95%		
Williams	30.9	±	1.8		255.0	±	13.4	
DPM25	31.5	±	2.0		244.0	±	15.0	
Dwarf Nathan	28.7	±	1.5		111.0	±	12.0	*
GCTCV 106	46.2	±	3.0	*	253.0	±	21.3	
GCTCV 215	38.8	±	1.5	*	268.0	±	12.1	
GCTCV 218	37.3	±	1.7	*	279.0	±	13.0	
GCTCV 247	35.0	±	1.6		260.0	±	12.0	
CJ19	34.2	±	1.5		207.0	±	12.0	*
FHIA-01	31.6	±	1.5		311.0	±	12.0	*
FHIA-02	28.0	±	1.5		261.0	±	11.8	
FHIA-03	35.5	±	1.5	*	307.0	±	11.8	*
FHIA-18	32.3	±	1.5		309.0	±	12.0	*
FHIA-25	45.0	±	1.5	*	321.0	±	12.0	*
SH-3142	36.1	±	1.8	*	321.0	±	13.4	*
SH-3217	40.6	±	1.5	*	356.0	±	12.0	*
SH-3362	48.9	±	1.5	*	353.0	±	12.0	*
SH-3436	36.7	±	1.5	*	316.0	±	12.0	*
SH-3641	28.3	±	1.5		302.0	±	11.8	*
SH-3656	33.6	±	1.7		282.0	±	13.0	
SH-3748	31.5	±	1.6		326.0	±	12.0	*
Dwarf Ducasse	39.7	±	1.6	*	282.0	±	12.5	
Pisang Gajih Merah	31.7	±	1.5		402.0	±	11.8	*

**Table 5:** Plant crop times and bunch weights of surviving cultivars. Error value for each cultivar is represented by the confidence interval set at 95% (CL 95%) for the mean, an asterisk indicates the mean is significantly different to Williams control (*p* < 0.05).

	Crop Cycle				Bunch Weight			
Cultivar	Weeks	CL 95%			kg	CL 95%		
Williams	45.0	±	2.6		23.9	±	5.8	
D. Nathan	39.5	±	1.2	*	15.9	±	2.9	*
GCTCV 215	51.0	±	1.2		21.6	±	3.0	
GCTCV 218	46.6	±	1.3		27.1	±	3.2	
GCTCV 247	46.4	±	1.2		20.9	±	3.0	
CJ19	45.1	±	1.1		21.3	±	2.9	
FHIA-01	45.6	±	1.1		35.4	±	2.9	*
FHIA-02	43.2	±	1.2		23.9	±	3.0	
FHIA-03	48.9	±	1.2		30.7	±	3.0	*
FHIA-18	49.2	±	1.1		23.4	±	3.0	
FHIA-25	55.9	±	1.2	*	30.9	±	3.0	*
SH-3142	49.7	±	1.4		24.9	±	3.4	
SH-3362	61.2	±	1.2	*	27.9	±	2.9	
SH-3217	53.5	±	1.2	*	21.0	±	3.0	
SH-3436	47.1	±	1.3		31.9	±	3.0	*
SH-3641	43.3	±	1.4		27.9	±	3.3	
SH-3656	46.9	±	2		18.9	±	4.6	
SH-3748	42.5	±	1.2		32.6	±	3.0	*
D. Ducasse	53.5	±	1.3	*	18.1	±	3.3	
P. Gajih Merah	49.3	±	1.2		33.8	±	2.9	*

## Data Availability

The data supporting the findings of this study are presented within this article and Supplementary Figures. Any additional data can be available on request to the corresponding author.

## Supplementary Materials

The following are available online at https://www.mdpi.com/article/10.3390/jof7080627/s1, Figure S1: Average time to external symptom appearance caused by *Foc* TR4 infection, Figure S2: Presence of external and internal symptoms on the two most susceptible cultivars, Figure S3: PCR amplification of products from pure *Foc* TR4 cultures isolated from pseudostems of cultivars showing signs of infect.
