# Supplementary material for: Banana Cultivar Field Screening for Resistance to Fusarium oxysporum f.sp. cubense Tropical Race 4 in the Northern Territory
Source: J Fungi (Basel). 2021 Aug 1;7(8):627. doi: 10.3390/jof7080627 (PMC8400552; doi:10.3390/jof7080627)
Supplement: Supplementary file 1 [file jof-07-00627-s001.zip › jof-1260439-supplementary.pdf]

Journal of Fungi

a

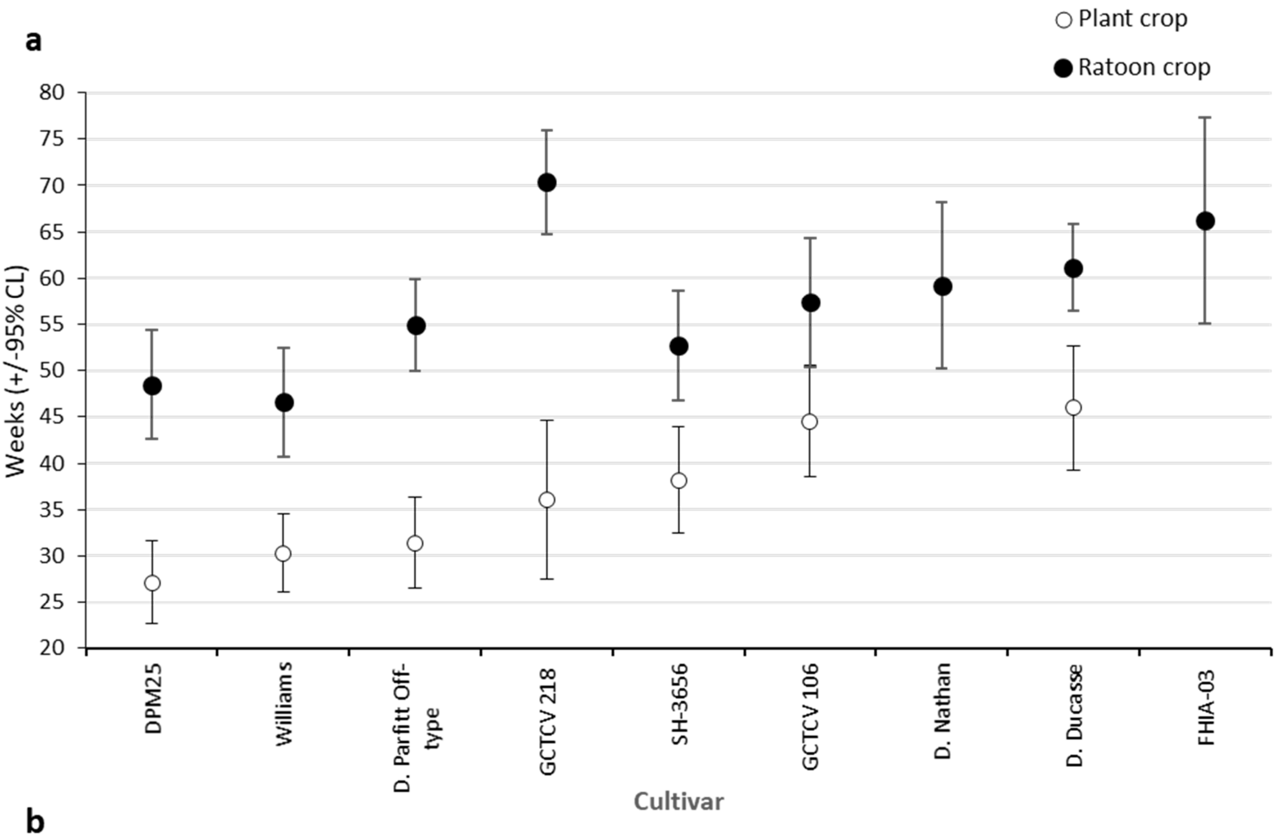

b

	DPM25	Williams	D. Parfitt Off-type	GCTCV 218	SH-3656	GCTCV 106	D. Nathan	D. Ducasse	FHIA-03
Plant crop	11	12	9	3	7	6	0	5	0
Ratoon crop	7	7	10	8	7	5	3	11	2

Figure S1: Average time to external symptom appearance caused by *Foc* TR4 infection. **a)** Time to external symptom appearance over two crop cycles. **b)** n values for corresponding data points shown in a). Error bars for each cultivar represent the 95% confidence interval for the mean.

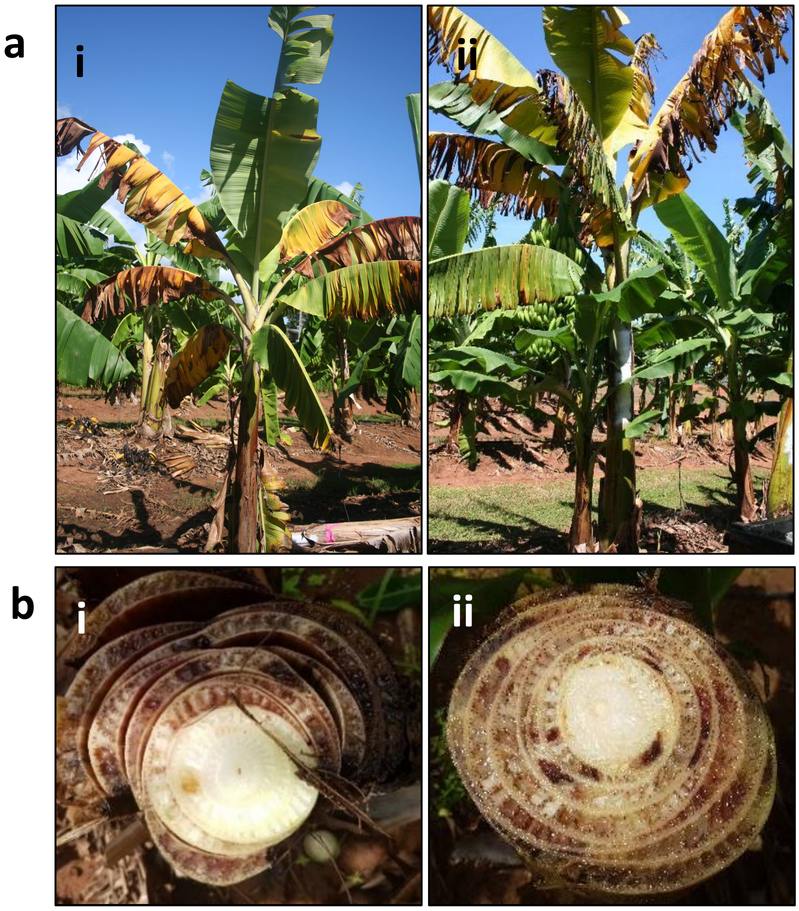

Figure S2: Presence of external and internal symptoms on the two most susceptible cultivars i) Williams and ii) DPM25. Symptoms noted in susceptible cultivars included **a)** leaf chlorosis and necrosis and **b)** the presence of internal symptoms within the pseudostems.

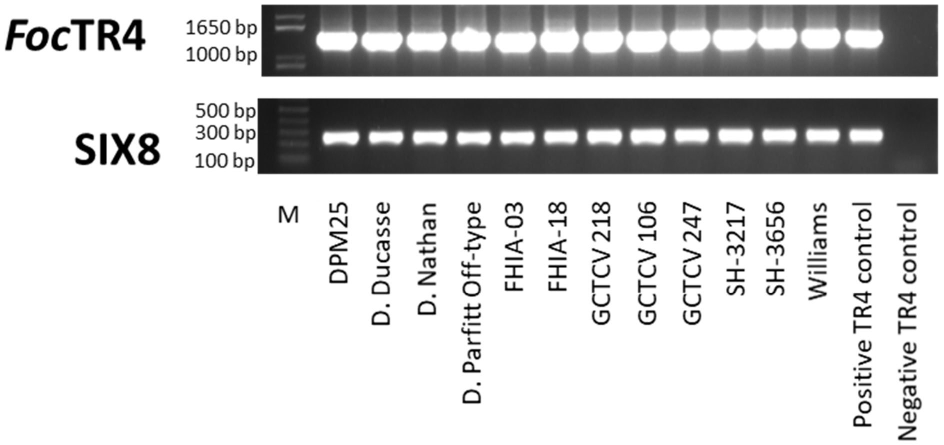

Figure S3: PCR amplification of products of pure *Foc* TR4 cultures isolated from pseudostems of cultivars showing signs of infection. M indicates the use of a molecular marker 1Kb DNA plus ladder.
